# What factors influence HIV testing? Modeling preference heterogeneity using latent classes and class-independent random effects

**DOI:** 10.1016/j.jocm.2021.100305

**Published:** 2021-07-11

**Authors:** Jan Ostermann, Brian P. Flaherty, Derek S. Brown, Bernard Njau, Amy M. Hobbie, Tara B. Mtuy, Max Masnick, Axel C. Mühlbacher, Nathan M. Thielman

**Affiliations:** a Department of Health Services Policy & Management, University of South Carolina, 915 Greene Street, Columbia, SC, USA; b South Carolina Smart State Center for Healthcare Quality, University of South Carolina, Columbia, SC, USA; c Duke Global Health Institute, Duke University, Durham, NC, USA; d Center for Health Policy & Inequalities Research, Duke University, Durham, NC, USA; e Department of Psychology, University of Washington, Seattle, WA, USA; f Brown School, Washington University in St. Louis, St. Louis, MO, USA; g Kilimanjaro Christian Medical Centre, Moshi, Tanzania; h Department of Global Health and Development, London School of Hygiene and Tropical Medicine, London, UK; i Selway Labs, LLC, Englewood, CO, USA; j Institut Gesundheitsökonomie und Medizinmanagement, Hochschule Neubrandenburg, Neubrandenburg, Germany; k Department of Population Health Sciences, Duke University, Durham, NC, USA

**Keywords:** Discrete choice experiment, Preference heterogeneity, Random effects latent class logit (RELCL), HIV counseling and testing, Tanzania

## Abstract

Efforts to eliminate the HIV epidemic will require increased HIV testing rates among high-risk populations. To inform the design of HIV testing interventions, a discrete choice experiment (DCE) with six policy-relevant attributes of HIV testing options elicited the testing preferences of 300 female barworkers and 440 male Kilimanjaro mountain porters in northern Tanzania. Surveys were administered between September 2017 and July 2018. Participants were asked to complete 12 choice tasks, each involving first- and second-best choices from 3 testing options. DCE responses were analyzed using a random effects latent class logit (RELCL) model, in which the latent classes summarize common participant preference profiles, and the random effects capture additional individual-level preference heterogeneity with respect to three attribute domains: (a) privacy and confidentiality (testing venue, pre-test counseling, partner notification); (b) invasiveness and perceived accuracy (method for obtaining the sample for the HIV test); and (c) accessibility and value (testing availability, additional services provided). The Bayesian Information Criterion indicated the best model fit for a model with 8 preference classes, with class sizes ranging from 6% to 19% of participants. Substantial preference heterogeneity was observed, both between and within latent classes, with 12 of 16 attribute levels having positive and negative coefficients across classes, and all three random effects contributing significantly to participants’ choices. The findings may help identify combinations of testing options that match the distribution of HIV testing preferences among high-risk populations; the methods may be used to systematically design heterogeneity-focused interventions using stated preference methods.

## 1. Introduction

Ambitious targets have been set by the Joint United Nations Programme on HIV/AIDS (UNAIDS), the President’s Emergency Plan for AIDS Relief (PEPFAR), and by Ministries of Health across the globe to eliminate the HIV epidemic. For the year 2030, these targets include what is known as 95–95-95 - diagnosing 95% of all persons living with HIV (PLWH), initiating treatment for 95% of those diagnosed, and achieving viral suppression for 95% of those treated (UNAIDS, 2014). Progress towards diagnosing 95% of PLWH by 2030 is contingent on accelerating the uptake of HIV testing, both among higher-risk populations and across the population at large. The number of undiagnosed HIV infections is considered a major hindrance to achieving the UNAIDS targets and ending the epidemic (The Lancet, 2017).

Discrete choice experiments (DCEs) are commonly used to elicit information about individuals’ preferences for varying characteristics of multi-attribute products. DCE results can be used to develop targeted, preference-informed interventions; optimal interventions may vary across and within population subgroups. DCEs have been used in various contexts related to HIV, including testing (Indravudh et al., 2017; Johnson et al., 2010; Ostermann et al., 2014, 2015; Phillips et al., 2002; Strauss et al., 2018a, 2018b), prevention (Cameron et al., 2013; Newman et al., 2016; Quaife et al., 2018a; Terris-Prestholt et al., 2013), service delivery (d’Elbee et al., 2018; Kruk et al., 2016; Zanolini et al., 2018), and treatment (Beusterien et al., 2007; Bregigeon-Ronot et al., 2017; Hauber et al., 2009; Ostermann et al., 2020a; Mühlbacher et al., 2013). We previously characterized the HIV testing preferences in a community sample in Tanzania and identified substantial preference heterogeneity (Ostermann et al., 2014). To our knowledge, DCEs have not been used to systematically characterize the distribution of HIV testing preferences among populations at high risk of HIV infection.

To inform the design of HIV testing interventions for high-risk populations, this study used a DCE to characterize patterns of testing preferences among female barworkers and male Kilimanjaro mountain porters, two high-risk populations in northern Tanzania. The DCE focused on policy-relevant characteristics of HIV testing programs that may be adapted to match the preferences of these populations. To identify patterns of preferences, we modeled DCE responses using a random effects latent class logit (RELCL) model. RELCL models allow the simultaneous estimation of common preference profiles via latent classes, as well as class-independent individual variation via random effects (Zhou and Bridges, 2019; Greene and Hensher, 2013; Hess et al., 2014). The findings from this study may help identify combinations of testing options that match the distribution of HIV testing preferences among the two high risk populations included in this study. More generally, the analytic approach described here may inform the systematic design of interventions in the context of preference heterogeneity.

## 2. Methods

### 2.1. Ethical approval

The study protocol was approved by the Institutional Review Boards at Duke University and the University of South Carolina in the United States, as well as the Ethics Review Committee at Kilimanjaro Christian Medical University College and the National Institute for Medical Research in Tanzania. The protocol was registered in ClinicalTrials.gov (Protocol NCT02714140) on March 21, 2016 (Ostermann et al., 2020). Informed consent was obtained from all study participants.

### 2.2. Study setting

The study was conducted in Moshi, Tanzania. Moshi is the commercial center and administrative capital of the Kilimanjaro Region in Northern Tanzania and has an estimated population of about 200,000 (United Republic of Tanzania, 2012). Voluntary HIV counseling and testing (VCT) is available at 25 health facilities, including 2 free-standing VCT centers.

### 2.3. Study sample

Study participants were enrolled between September 2017 and July 2018. Participants comprised 300 women employed in bars, restaurants and guesthouses serving alcohol to patrons (henceforth referred to as “bars” and “female barworkers”, respectively) and 440 male mountain porters supporting climbers of nearby Mount Kilimanjaro (“male porters”). We previously showed that female barworkers and male porters engage in higher rates of HIV risk behaviors than randomly selected male and female community members in the same setting (Ostermann et al., 2015). A census of bars and barworkers, conducted by the study team between February and June of 2016, identified 612 bars within Moshi, with 2059 age-eligible female barworkers. There are an estimated 10, 000 porters in the Kilimanjaro Region (Mitchell et al., 2009; Peaty, 2012).

Eligible study participants were residents of Moshi, able to read, and ages 18 to 49. Female barworkers were recruited from randomly selected bars; male porters were sequentially approached as they exited Mount Kilimanjaro National Park. Eligible individuals were invited to the study’s research office for consent and enrollment; study compensation ranged from Tanzania Shilling (TSH) 5000 (~$2.15) to TSH 10,000 (~$4.30), including transport reimbursement.

### 2.4. Discrete choice experiment

HIV testing preferences were assessed using a DCE. The objective of the DCE was to present survey respondents with hypothetical HIV testing options that could feasibly be implemented in the study area. As such, the DCE was built on the characteristics of testing options that were available in the study area at the time of the survey, as well as characteristics that could feasibly be implemented. The design, administration, and analysis of the DCE followed the guidelines for DCE applications in healthcare (Bridges et al., 2011) and in low-income settings (Mangham et al., 2009). As with a previous DCE on HIV testing preferences in the same area, the selection of attributes was guided by focus group discussions with members of the target populations (Ostermann et al., 2014, 2015; Njau et al., 2014). The attributes and levels employed in our study were the following:

#### Attribute 1 – Testing venue.

In the study area, HIV testing is available at health facilities and at free-standing VCT centers. Several of these facilities also conduct outreach activities, including home-based testing, which involves a counselor coming to a client’s home for VCT. Testing venue was thus implemented as a three-level attribute: testing at a *health facility*, testing at a free-standing *VCT center*, and testing at *home*.

#### Attribute 2 – Testing availability.

The majority of testing venues in Moshi offer HIV testing only on weekdays. However, selected facilities started making testing available on weekends. In the DCE, testing availability was implemented as a two-level, ordered, attribute describing testing availability on either *weekdays only* or *every day of the week*.

#### Attribute 3 – Pre-test counseling.

National testing guidelines (National AIDS Control Programme, 2019) require that, before testing for HIV, a counselor provides the client with information about HIV, risk of infection, and the HIV test. This service is referred to as *pre-test counseling*. Pre-test counseling can be done one-on-one, in a group, or with a partner in the context of couples counseling and testing. Accordingly, this attribute was implemented as a three-level attribute.

#### Attribute 4 – Type of sample.

Three different methods may be used to collect the sample for the HIV test. Samples can be collected using blood from the arm (*venipuncture*) or the finger (*finger prick*), or saliva can be taken from the mouth using an *oral swab*. Accordingly, the attribute was implemented as a three-level attribute. It was emphasized that all three options give the same result; however, oral testing was not yet approved for general use in Tanzania at the time of the survey.

#### Attribute 5 – Additional services.

To decrease stigma and increase value, there have been efforts to integrate HIV testing with other health services (National AIDS Control Programme, 2017). This attribute was implemented as a three-level attribute describing the provision of additional services in conjunction with the HIV test, namely a complimentary *screen for other sexually transmitted infections (STIs)*, a complimentary general *health check* (e.g., blood pressure, diabetes), versus *no additional services.*

#### Attribute 6 - Partner notification.

For persons testing positive for HIV, the notification and testing of sexual partners is critical for identifying or preventing additional HIV infections (Brown et al., 2011; Cherutich et al., 2017; Chiou et al., 2015; Garcia de Olalla et al., 2015; Henley et al., 2013; Kahabuka et al., 2017; Landis et al., 1992; Myers et al., 2016; Rosenberg et al., 2015; Udeagu et al., 2012, 2014). Partner notification was implemented as a three-level attribute. *Self-disclosure* involves clients testing positive being encouraged to advise their partners to test for HIV. Confidential *provider notification* involves clients being asked to give the name and contact information for their partners, and a counselor later contacting these partners to test for HIV without revealing the client’s name. *Automatic disclosure* involves the joint receipt of HIV test results by clients and their partners in the context of couples counseling.

#### 2.4.1. Experimental design

We measured preferences over the full range of feasible combinations of attribute levels by presenting respondents with a range of HIV testing options, based on an experimental design, and observing their stated preferences. The experimental design of a DCE is the combination of choice tasks that allows for the independent estimation of the influence of each testing characteristic on preferences. Ngene software (ChoiceMetrics, 2017) version 1.12b was used to select an experimental design that minimized the D-error for a mixed logit model (Johnson et al., 2007). Two constraints were imposed in the selection of choice tasks for the experimental design:

1. To exclude non-feasible combinations of attribute levels for the pre-test counseling and partner notification attributes (e.g., *couples counseling* with *self-disclosure*), these two attributes were combined into a 5-level compound attribute. Four levels of the compound attribute described combinations of either *one-on-one* or *group counseling* with either *self-disclosure* or confidential *provider notification*; the fifth level described *couples counseling with automatic partner notification.*
2. Statistical priors were obtained from a pilot study with 236 female barworkers and male porters. Data were analyzed using a mixed logit model; the estimated means and standard deviations were used as priors in the search for a D-efficient design optimized for a mixed logit model.

The final design consisted of 120 tasks. Participants were randomized across 10 blocks with 12 tasks each. The order of choice tasks in a block was randomized across participants. Each choice task included three unlabeled testing alternatives; the order of alternatives was randomized within each choice task.

#### 2.4.2. DCE administration

**Fig. 1.**
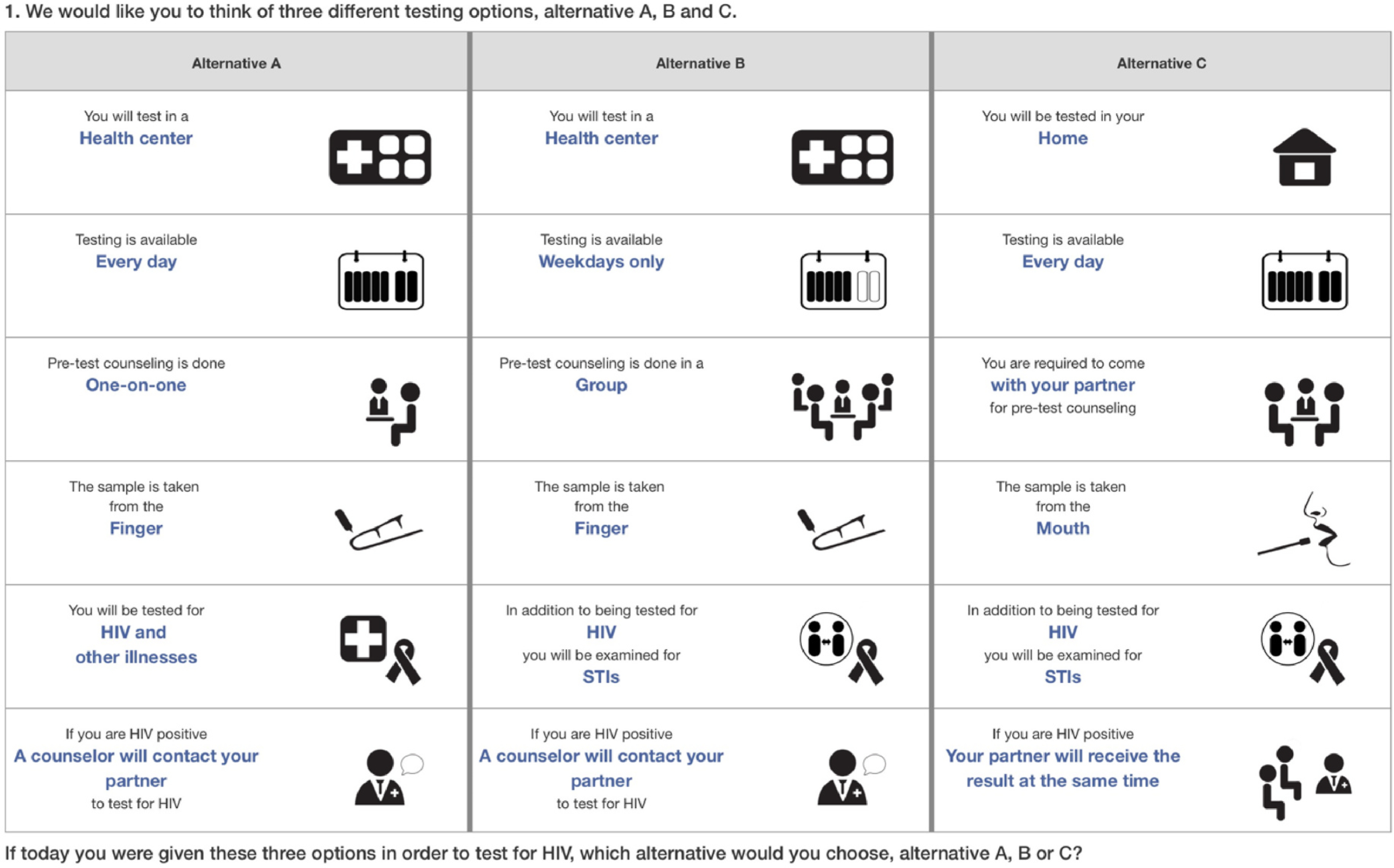
Sample DCE choice task.

In-person DCE surveys were fielded by trained research staff, in Kiswahili (a language commonly used in the study area), on iPad devices, using *Comet* survey software (Selway Labs, 2017). Participants initially ranked the levels of each attribute. These data were used to populate a respondent-specific comprehension task with clearly dominant (preferred levels for all attributes) and dominated (worse levels for all attributes) alternatives, followed by 12 DCE choice tasks. In each choice task, participants were first asked to select their most preferred option from three testing options presented; in a follow-up task they were asked to select their most preferred from the two remaining options. A sample choice task is shown in Fig. 1.

### 2.5. Supplemental survey

A supplemental survey assessed sociodemographic characteristics and the HIV testing history of study participants.

### 2.6. Econometric model

Respondents’ rankings of the HIV testing options presented in the DCE choice tasks were modeled with a latent class conditional logit model with three individual-level, class independent, random effects. Our model did not include alternative-specific constants because choices were unlabeled and presented in random order. Let *i* index respondents and *t* index choice tasks (1 ≤ t ≤ T = 12). Further, let *y*_*it*_ = *m* denote respondent *i*’s choice of option *m* in task *t*, and let *M* = *3* denote the number of alternatives in each ranking task. The probability of response *m* based on the basic latent class conditional logit model, without random effects, is

$$P\left(y_{it}=m|x,z_{itm}\right)=\frac{\exp\left(\eta_{m}|x,z_{\dot{u}m}\right)}{\sum_{j=1}^{M}\exp\left(\eta_{j}|x,z_{itj}\right)},$$

where *x* denotes latent class membership, ***z***_***itm***_ denotes the vector of attribute levels associated with alternative *m* for respondent *i* in task *t;* and *η*_*m*_|x,z_***itm***_ denotes the utility associated with alternative *m* conditional on membership in latent class *x* and attribute levels ***z***_***itm***_. The linear model for the utility of alternative *m* characterized by attributes ***z***_***itm***_ for a member of latent class *x* is

$$\eta_{m}|x,z_{itm}=\sum_{p=1}^{P}\beta_{xp}z_{itmp},$$

where *p* indexes attribute levels of alternative *m* (*p* = *1, …, P)*; *β*_*xp*_ is the regression coefficient for attribute level *p* for latent class *x*; and *z*_*itmp*_ is the effects coding for attribute level *p* in alternative *m* for individual *i* in task *t*.

Let ***F***_***i***_ denote individual *i*’s vector of *d* (*d* = *1, …, D)* scores *on D* independent and standard normal random effects distributions. Adding ***F***_***i***_ to the model, the probability of respondent *i* making choice *m* is

$$P\left(y_{\dot{it}}=m|x,z_{itm},F_{i}\right)=\frac{\exp\left(\eta_{m}|x,z_{itm},F_{i}\right)}{\sum_{j=1}^{M}\exp\left(\eta_{j}|x,z_{itj},F_{i}\right)},$$

and the utility function is

(1) $$\eta_{m}|x,z_{itm},F_{i}=\sum_{p=1}^{P}\beta_{xp}z_{itmp}+\sum_{d=1}^{D}\sum_{p=1}^{P}\lambda_{dp}F_{id}z_{itmp},$$

where *F*_*id*_ is the random effect *d* for individual *i*, and *λ*_*dmp*_ is a coefficient (i.e., loading) relating random effect *F*_*id*_ and attribute level *z*_*itmp*_ to the utility of alternative *m*.

The specific model estimated here includes *D* = *3* subject level random effects that capture individual-level preference heterogeneity with respect to the following attribute domains: (a) privacy and confidentiality (*F*_*.1*_, covering the *testing venue*, *pre-test counseling*, and *partner notification* attributes), (b) invasiveness and perceived accuracy (*F*_*.2*_, covering the *type of sample* attribute), and (c) accessibility and value (*F*_*.3*_, covering the testing availability and additional services attributes). These domains correspond to the three pillars of a conceptual model of preference-relevant HIV testing characteristics that we previously developed using qualitative work in the study area (Njau et al., 2014). The influence of the random effects (i.e., *F*_*.1*_*, F*_***.2***_*, F*_***.3***_) on the probability of choice *m* is determined by the corresponding λ weights. The product term (i.e., the third term in Equation (1)) modifies the latent class-specific preference term (i. e., the second term in Equation (1)) for the individual. Thus, this model accounts for preference heterogeneity in two ways: latent classes, which capture heterogeneity in preference profiles that are shared among groups of individuals (i.e., combinations of preferences that are likely to co-occur), and additional individual-level heterogeneity in the form of domain specific random effects that capture respondents’ unique attribute preferences, independent of class. Class membership is modeled as a function of only one covariate, participant type (barworkers vs. porters).

### 2.7. Statistical analysis

Differences in sociodemographic characteristics and HIV testing experiences between the two study cohorts were analyzed using Student’s *t*-tests and chi-squared statistics. The DCE data, composed of rankings of the three testing options presented to participants in each choice task, were analyzed as sequential choices: following an initial choice of the most preferred of the three options presented, a second choice involved the selection of the more preferred of the two remaining options. Random effects latent class logit (RELCL) models with 1–10 preference classes and 0 to 3 continuous, normally distributed random effects were estimated in Latent Gold Choice version 5.0 (Statistical Innovations Inc. 2018). Models were estimated using expectation-maximization (EM) and Newton–Raphson (NR) algorithms, with 250 EM and 50 NR iterations, and 16 different sets of random starting values. The Bayesian Information Criterion (BIC) was used to compare model fit. The best-fitting model was re-estimated with 150 different sets of random starting values to check that a global optimum was obtained. For the final model, correlates of class membership were evaluated using the bias-adjusted three-step approach described by Bakk et al. (2014). Additionally, separate models with the same specification were estimated for each risk group, and class membership predictions, based on modal estimated class membership probabilities, were compared between the aggregate and cohort-specific models.

## 3. Results

Table 1 details key demographic characteristics and the HIV testing history of study participants. Approximately half of the participants had at least some secondary school education, and most participants had tested for HIV at least once. Female barworkers were less likely to be married, had higher education, and were more likely to have tested for HIV than male Kilimanjaro mountain porters. Compared to a national sample of adults ages 18–49 residing in mainland urban Tanzania who participated in the 2016–17 Tanzania HIV IMPACT Survey (THIS) (Tanzania Commission for AIDS, 2018), female barworkers were less likely to be married, had more education, and both groups were somewhat more likely to have ever tested for HIV.

**Fig. 2.**
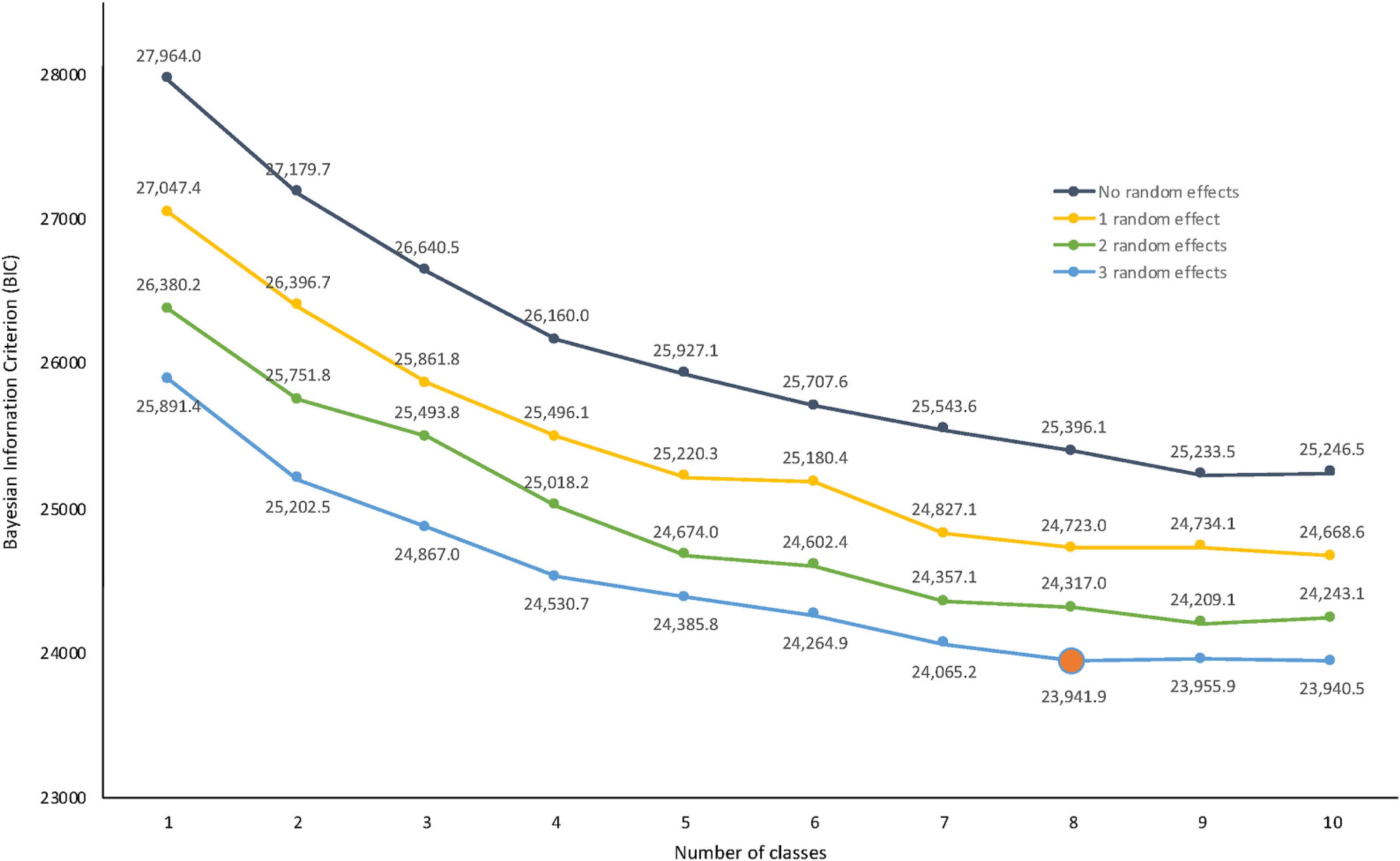
Relative performance of alternative latent class specifications with 0–3 random effects.

Fig. 2 shows the performance of latent class logit (LCL) models with and without random effects specifications, as measured by the BIC. The RELCL models consistently outperformed LCL models without random effects, with model performance improving with additional random effects. Comparisons of the BIC across models indicated that, among models with 3 random effects, model fit continuously improved up to 8 classes, with only marginal improvements gained form additional classes.

Note: The orange marker indicates the model presented below, selected based on the model’s relative performance on the Bayesian Information Criterion (BIC).

Table 2 shows the results of the RELCL model with 8 preference classes and 3 random effects. As with a standard latent class model, the 8 preference classes represent statistical groupings of individuals with similar sets of preferences. Unlike a standard latent class model, with a RELCL model, the three random effects capture additional class-independent individual preference heterogeneity with respect to the five attributes grouped broadly into three domains: privacy and confidentiality (testing venue; pre-test counseling; partner notification); invasiveness and perceived accuracy (type of sample); and accessibility and value (testing availability; additional services).

The data included 17760 choices of 740 participants, totaling 8880 rankings (12 best and second-best choices per participant yield 12 rankings * 740 participants = 8880 rankings). The latent preference classes range in size from 6% to 19% of participants; distributions are similar between female barworkers and male porters.

Class-specific preferences for each attribute level included in the DCE are described by the estimated (effects coded) coefficients. Results indicate substantial heterogeneity; the variation in parameters within and across classes, combined with a comparatively large number of classes, preclude the labeling of classes based on patterns of coefficients. All attribute levels except testing availability (which was constrained to be ordered), oral testing, and HIV testing only (without additional services) have positive and negative coefficients across classes, indicating that they are preferred by some groups of participants (classes) and disliked by others. The largest coefficient ranges across preference classes were observed for a health check alongside the HIV test, couples counseling with automatic disclosure of a positive HIV test result, the different testing venues, and preferences for venipuncture or finger prick.

The final column in Table 2 shows the effects of the random effects on utility. The estimated parameters (“loadings”) describe the extent to which the random effects amplify (same sign for the class-specific utility weight and the loading) or offset (different signs for the class-specific utility weight and the loading) the class-specific effect of each attribute level on utility. The effects differ greatly across attribute levels. The largest loadings were observed for (a) couples counseling and testing with automatic disclosure of a positive HIV test result, (b) oral swabs, and (c) venipuncture. The estimates indicate that for 3 out of 6 classes in which couples counseling was positively associated with utility, a difference of one standard deviation in random effect 1 more than offsets these utility gains. Similarly, a difference of one standard deviation in random effect 2 more than offsets the (average) aversion to oral swabs for all 8 classes and more than doubles the positive effect of venipuncture on utility among 4 out of 6 preference classes.

**Fig. 3.**
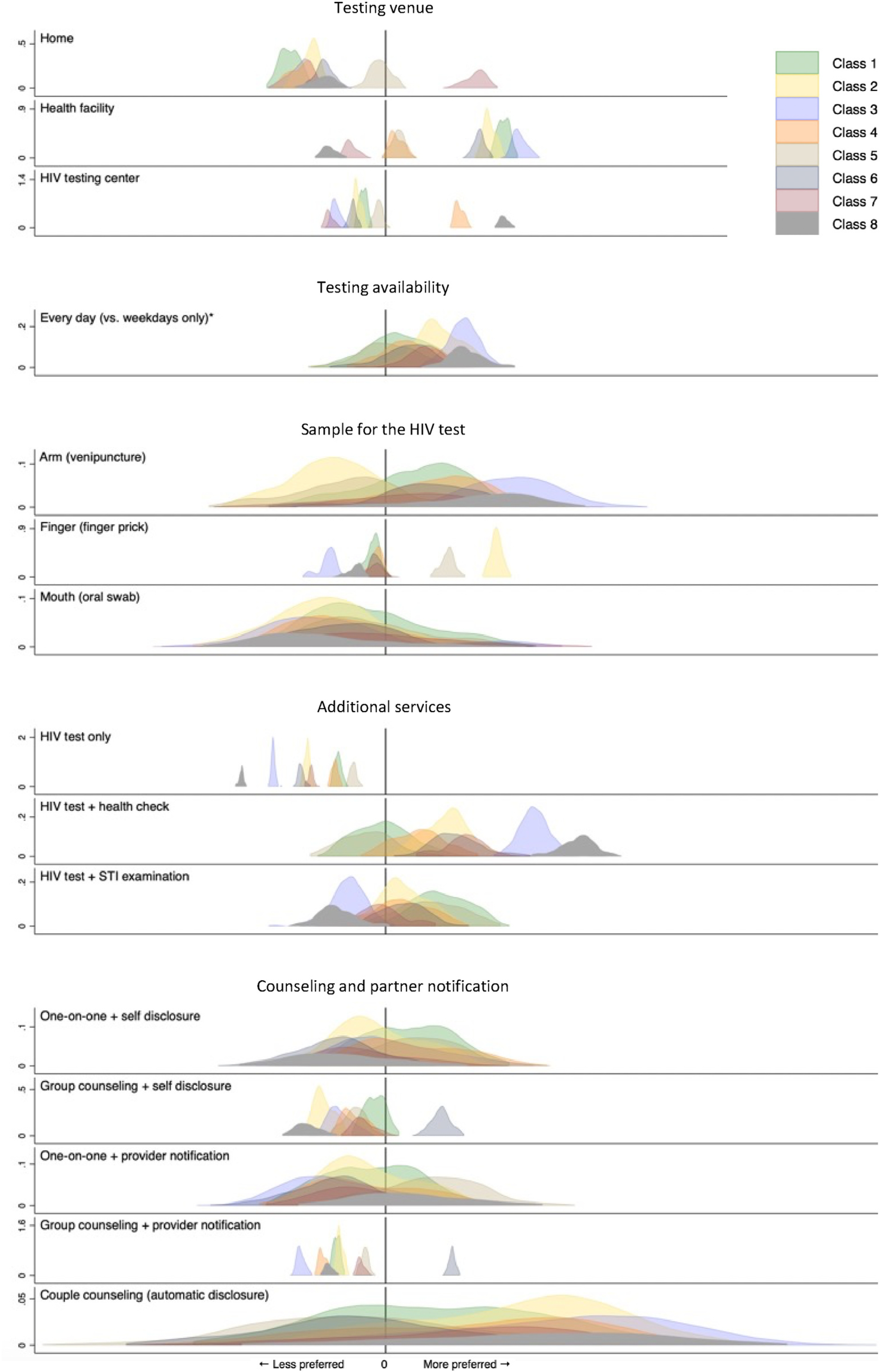
Visualization of between- and within-class preference heterogeneity; estimates from a random effects latent class logit model (N = 740) Notes: Distributions represent kernel densities of individual-level preference estimates conditional on modal class membership probability and individuals’ posterior scores on three domain-specific random effects. Each color represents one preference class. Within attribute levels, kernel densities were scaled in proportion to class size; y-axis scales vary across attribute levels. * Estimates for the *weekdays only* attribute level are symmetric around x = 0. (For interpretation of the references to color in this figure legend, the reader is referred to the Web version of this article.)

Given that the random effects have standard normal distributions, the absolute values of the loadings also characterize the variability of (class-specific) preference estimates across individuals. This individual-level heterogeneity, alongside the heterogeneity in preference profiles described by the latent classes, is visualized in Fig. 3. The distributions describe individual level relative preferences for (positive values) or aversion against (negative values) the respective characteristics evaluated in the DCE, conditional on predicted class membership (which is represented by different colors). The largest absolute loading in Table 2 (the λ value for couples counseling and testing with automatic disclosure) corresponds to the widest distributions of individual level preference estimates around class-specific means.

Table 3 documents systematic variation in estimated class membership probabilities with demographic characteristics and HIV testing history. Older, less educated, never testers were more likely to be members of Class 1; frequent testers were most likely to be members of classes 7 and 8. Whilst gender, and thus risk group, was associated with class membership (e.g., male porters were less likely to be members of classes 1, 3, and 4), the corresponding parameter estimates were smaller than those of variables describing testing history. Age, education, marital status, and testing history were significantly associated with the random effects for the confidentiality and privacy domain (λ_1_), while education and a prior HIV test were associated with the random effect for the invasiveness and perceived accuracy domain (λ_2_). Strong concordance was observed between study participants’ groupings into preference classes based on gender-specific vs. aggregate RELCL models (Appendix 1).

## 4. Discussion

In this study of the HIV testing preferences of 300 female barworkers and 440 male Kilimanjaro mountain porters, two high-risk populations in Northern Tanzania, we identified substantial preference heterogeneity across individuals. Our findings provide strong support for the provision of an array of diverse HIV testing options in the study area that target the heterogeneous testing preferences of high-risk populations.

To our knowledge this study is the largest DCE of preferences for HIV counseling and testing focused on high-risk populations and on policy-relevant testing attributes. In addition, this study is the first to specifically focus on preference heterogeneity. While prior studies, including our own (Ostermann et al., 2014, 2015), have documented preference heterogeneity, the analysis of sources of heterogeneity was limited to systematic variation in mean preference parameters between population subgroups identified on the basis of covariates. This study uses a RELCL model to jointly characterize heterogeneity in preference profiles that are shared among groups of respondents (i.e., combinations of preferences that are likely to co-occur among the two high-risk populations) and additional individual-level heterogeneity that captures respondents’ unique attribute preferences, independent of class. Specifically, the latent classes capture some of the correlations among all the estimated part-worth utilities. Additionally, the three random effects, each linked to an attribute domain, and the corresponding loadings, further describe individual-level heterogeneity in the magnitude of the part-worth utilities and correlations among the part-worth utilities across attribute levels within attribute domains. Fig. 3 illustrates substantial variation in the distributions of the estimated preferences across attribute levels, classes, and individuals, thereby highlighting the distributional flexibility of the RELCL model employed in this study.

We acknowledge several limitations of the study. First, study participants were recruited from two high-risk populations in Northern Tanzania, and HIV testing options were described with only six characteristics. The number of attributes and levels presented could not cover all testing characteristics that might be important to a given participant. To ensure policy relevance, our selection of attribute levels was guided by actual and feasible characteristics across the 25 HIV counseling and testing providers in the study area. Other characteristics of HIV testing options may influence testing preferences and uptake in other settings and populations.

Second, while our study identified substantial preference heterogeneity, it was unable to discern the sources or consequences of this variation. Class membership probabilities and the distribution of random effects varied systematically with age, education, marital status, and HIV testing history (Table 3), however, the estimated distributions of the two distinct sub-populations across preference classes were nearly identical, there were no systematic differences in the distributions of random effects between barworkers and porters, and gender-specific models resulted in similar groupings of individuals as the aggregate model. While additional studies are needed to characterize the extent to which specific individual-level characteristics (e.g., knowledge and information, prior experiences with HIV testing, perceptions of HIV risk, anticipated consequences of a positive HIV test), correlate with preferences, our results suggest that a substantial share of preference heterogeneity may not be explainable by general demographic and risk characteristics. From a policy perspective, it may thus be more important to evaluate the extent to which heterogeneous population preferences align with the characteristics of existing testing options, and to explore associations with testing uptake among high-risk populations.

Third, we acknowledge several methodological limitations. These include the lack of experimental design software that would have allowed us to identify an experimental design optimized for a latent class model; the omission of interactions; and general limitations of DCEs, such as the potential for hypothetical bias (Quaife et al., 2018b).

## 5. Conclusion

This study describes substantial heterogeneity in preferences for HIV testing among two high-risk populations in Tanzania, including distinct preference profiles that are shared among groups of individuals, and additional, random variation across individuals. From a practical perspective, the study results provide strong support for the provision of an array of HIV testing options to target preference heterogeneity and maximize uptake of HIV testing among high-risk populations. The methods we describe may be applicable to other populations, settings, and choice contexts in which similar preference heterogeneity is suspected and can serve as a starting point for the systematic design of heterogeneity-focused interventions using stated preference methods.

## Figures and Tables

**Table 1:** Characteristics of study participants.

		Study sample^b^			National sample^c^	
				
		(Ages 18–49, urban)			(Ages 18–49, urban)	
				
		Female barworkers	Male porters		Females	Males

Number of participants		300	440	p-value	5067	4077
Age	Mean (sd)	29.7 (7.61)	31.4 (6.71)	0.001	29.6 (8.4)	29.8 (8.4)
Marital status	Married	31.8%	65.6%	<0.001	58.5%	65.9%
	Not married	68.2%	34.4%		41.5%	34.1%
Education	Primary school or less	42.1%	55.4%	<0.001	62.5%	51.8%
	Secondary school	57.9%	44.6%		37.5%	48.2%
# of HIV tests	None	5.3%	20.0%	<0.001	12.3%	26.6%
	1	13.7%	20.0%		.	.
	2	19.3%	23.4%		.	.
	3	26.3%	18.4%		.	.
	4	15.3%	6.8%		.	.
	5 or more	20.0%	11.4%		.	.
Most recent HIV test ^a^	In the past year	46.1%	49.4%	0.407	50.8%	51.5%
	More than 1 year ago	53.9%	50.6%		49.2%	48.5%

Notes.

Sample restricted to adults ages 18–49, living in mainland urban Tanzania; https://phia-data.icap.columbia.edu/files.

Information not available.

a Among those who tested at least once.

b Demographic data (age, marital status, education) are missing for 1 porter and 1 barworker

c Source: Survey-weighted means and percentages from the 2016–2017 Tanzania HIV Impact Survey (THIS).

**Table 2:** Heterogeneous HIV testing preferences: Results from a random effects latent class logit model (RELCL; N = 740).

		Class1		Class2		Class3		Class4		Class5		Class6		Class7		Class8			

**Class sizes**																			
All participants		18.9%		18.7%		13.5%		12.8%		12.5%		10.1%		7.5%		6.1%			
Female barworkers		20.0%		18.0%		15.4%		13.8%		12.6%		9.7%		4.6%		6.1%			
Male mountain porters		18.3%		19.1%		12.2%		12.1%		12.5%		10.3%		9.5%		6.1%			
	**Class-specific coefficient estimates, β**																	**Loadings, λ** ^a^	
**Testing venue**																			
	Home	−1.34	(0.09)	−1.06	(0.08)	−1.18	(0.09)	−1.19	(0.11)	−0.07	(0.08)	−0.84	(0.09)	1.26	(0.12)	−0.87	(0.13)	−0.168	1
	Health facility	1.65	(0.13)	1.45	(0.10)	1.87	(0.12)	0.16	(0.14)	0.17	(0.09)	1.30	(0.11)	−0.48	(0.10)	−0.78	(0.15)	0.103	1
	Voluntary counseling and testing center	−0.31	(0.10)	−0.39	(0.08)	−0.69	(0.10)	1.03	(0.17)	−0.10	(0.08)	−0.46	(0.10)	−0.78	(0.11)	1.65	(0.18)	0.065	1
**Testing availability**																			
	Weekdays only	−0.15	(0.07)	−0.72	(0.06)	−1.09	(0.08)	−0.35	(0.09)	0.00	(0.07)	−0.33	(0.09)	−0.61	(0.08)	−1.13	(0.13)	0.408	3
	Every day	0.15	(0.07)	0.72	(0.06)	1.09	(0.08)	0.35	(0.09)	0.00	(0.07)	0.33	(0.09)	0.61	(0.08)	1.13	(0.13)	−0.408	3
**Type of sample**																			
	Arm (venipuncture)	0.50	(0.11)	−0.66	(0.12)	1.45	(0.16)	0.64	(0.19)	−0.55	(0.13)	0.59	(0.14)	0.26	(0.17)	1.29	(0.21)	0.911	2
	Finger (finger prick)	−0.17	(0.08)	1.55	(0.10)	−0.81	(0.10)	−0.14	(0.11)	0.87	(0.08)	−0.15	(0.09)	−0.15	(0.09)	−0.42	(0.16)	0.114	2
	Mouth (oral swab)	−0.33	(0.12)	−0.89	(0.13)	−0.64	(0.14)	−0.50	(0.22)	−0.32	(0.13)	−0.44	(0.15)	−0.11	(0.18)	−0.87	(0.19)	−1.025	2
**Additional services**																			
	HIV test only (no additional services)	−0.66	(0.06)	−1.09	(0.08)	−1.57	(0.11)	−0.71	(0.10)	−0.45	(0.06)	−1.18	(0.09)	−1.04	(0.09)	−2.01	(0.16)	0.049	3
	Health check	−0.02	(0.08)	0.87	(0.09)	2.07	(0.15)	0.44	(0.11)	−0.22	(0.08)	0.96	(0.12)	1.10	(0.12)	2.68	(0.22)	0.391	3
	STI examination	0.68	(0.08)	0.22	(0.08)	−0.51	(0.09)	0.26	(0.10)	0.67	(0.09)	0.22	(0.10)	−0.06	(0.10)	−0.67	(0.14)	−0.440	3
**Pre-test counseling and partner notification**																			
	One-on-one counseling; self-disclosure	0.32	(0.10)	−0.08	(0.12)	0.07	(0.14)	0.37	(0.21)	0.38	(0.13)	−0.72	(0.15)	−0.30	(0.16)	−0.58	(0.22)	0.735	1
	Group counseling; self-disclosure	−0.14	(0.10)	−0.86	(0.10)	−0.63	(0.12)	−0.44	(0.11)	−0.43	(0.10)	0.76	(0.14)	−0.30	(0.13)	−1.09	(0.22)	0.175	1
	One-on-one counseling; provider notification	−0.18	(0.12)	−0.22	(0.15)	−0.62	(0.17)	−0.22	(0.24)	0.63	(0.15)	−0.72	(0.18)	−0.25	(0.17)	0.35	(0.26)	0.786	1
	Group counseling; provider notification	−0.68	(0.09)	−0.63	(0.09)	−1.18	(0.11)	−0.87	(0.13)	−0.29	(0.10)	0.92	(0.14)	−0.34	(0.11)	−0.79	(0.16)	0.059	1
	Couples counseling (automatic disclosure)	0.69	(0.20)	1.80	(0.28)	2.36	(0.30)	1.17	(0.45)	−0.28	(0.26)	−0.24	(0.29)	1.19	(0.29)	2.11	(0.38)	−1.755	1

Notes: Estimates from a RELCL model with 8 preference classes and 3 class-independent random effects.

a 1, 2, and 3 indicate the corresponding attribute domain: λ_1_ – privacy and confidentiality; λ_2_ – invasiveness and perceived accuracy; λ_3_ – accessibility and value.

**Table 3:** Correlates of latent class membership and individual-specific random effects (N = 740).

	Age			Some secondary school education			Married			Tested once			Tested more than once			Male porters		

Class 2	−0.08	***	(0.00)	−0.03		(0.05)	0.12	*	(0.06)	1.01	***	(0.09)	0.67	***	(0.08)	0.35	***	(0.06)
Class 3	−0.07	***	(0.00)	−0.49	***	(0.06)	0.02		(0.06)	0.88	***	(0.10)	0.62	***	(0.08)	−0.05		(0.06)
Class 4	−0.06	***	(0.00)	−0.29	***	(0.06)	0.10		(0.07)	0.16		(0.10)	−0.21	**	(0.08)	−0.13		(0.07)
Class 5	−0.02	***	(0.00)	−0.09		(0.06)	−0.48	***	(0.07)	0.41	***	(0.09)	−0.03		(0.07)	0.24	***	(0.06)
Class 6	−0.09	***	(0.00)	0.04		(0.07)	−0.13	*	(0.07)	0.78	***	(0.11)	0.64	***	(0.08)	0.45	***	(0.06)
Class 7	−0.05	***	(0.01)	−0.23	***	(0.06)	0.09		(0.07)	1.46	***	(0.12)	1.11	***	(0.11)	1.09	***	(0.08)
Class 8	−0.06	***	(0.01)	−0.52	***	(0.08)	−0.42	***	(0.08)	1.51	***	(0.13)	1.16	***	(0.11)	0.39	***	(0.07)
λ1	0.01	*	(0.01)	0.14	*	(0.07)	−0.29	***	(0.07)	−0.13		(0.11)	−0.32	**	(0.09)	−0.02		(0.07)
λ2	−0.01		(0.01)	−0.16	*	(0.07)	0.05		(0.07)	0.22	*	(0.11)	0.12		(0.10)	−0.09		(0.07)
λ3	0.00		(0.01)	−0.03		(0.07)	−0.05		(0.07)	0.19		(0.11)	−0.01		(0.09)	0.03		(0.07)

Notes: Coefficients and standard errors for correlates of class membership estimated using a bias-adjusted multinomial logit model; class 1 is the reference class; constants not shown. Coefficients and standard errors for correlates of individual-specific random effects estimated using linear regression models.

*, **, and *** indicate statistical significance at the 0.05, 0.01, and 0.001 levels, respectively.

**Figure:**
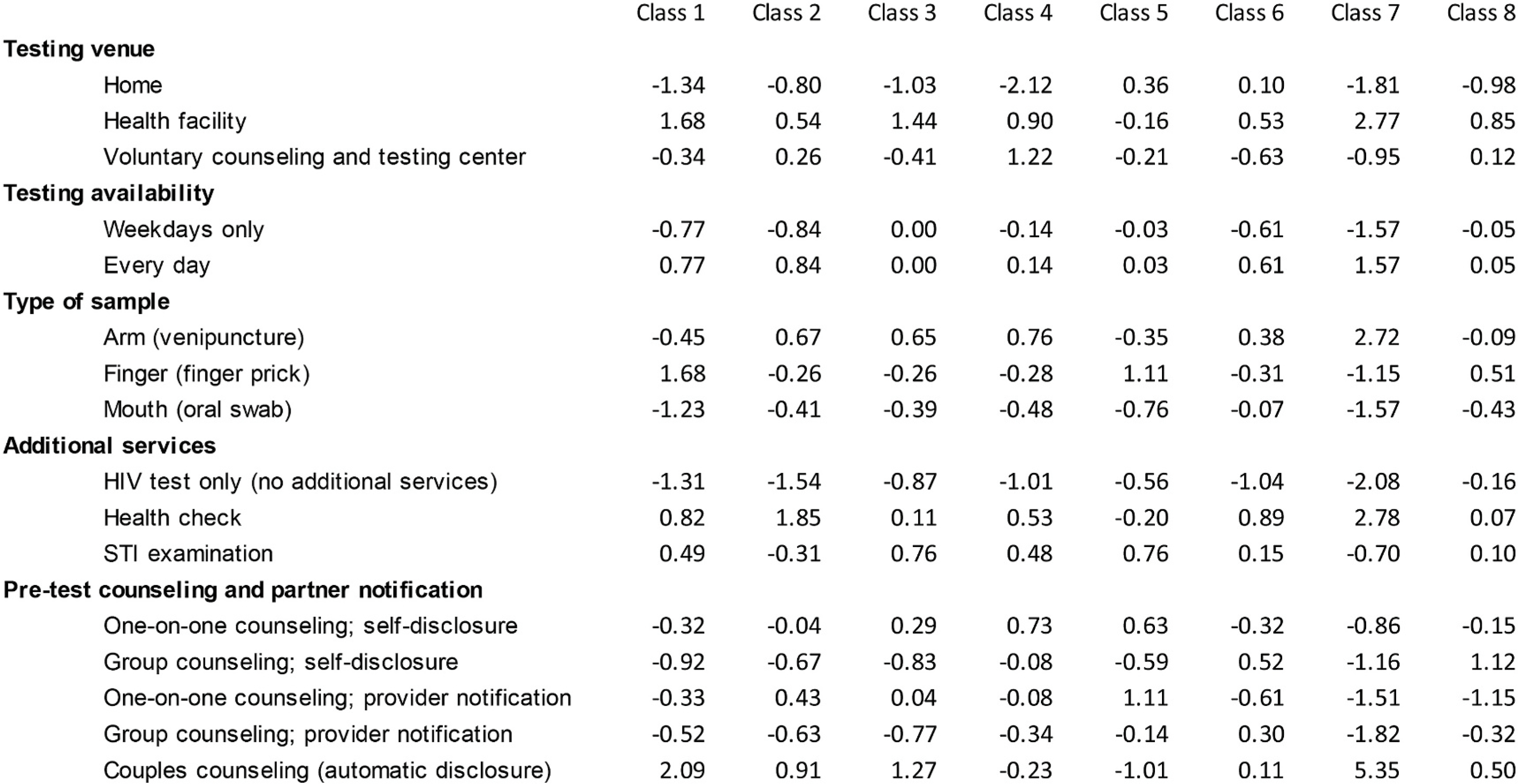
**Panel A.** Parameter estimates for female barworkers (N = 300).

**Figure:**
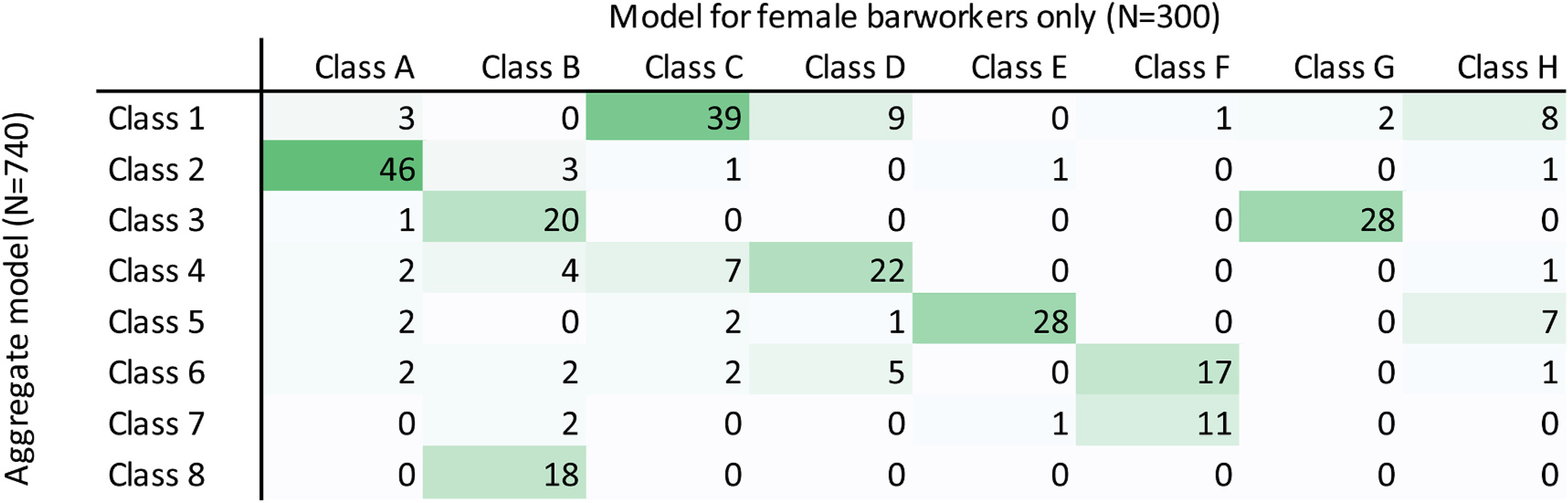
**Panel B.** Concordance between class membership predictions from gender-specific (N = 300 female barworkers only) vs. aggregate (N = 740) latent class models.

**Figure:**
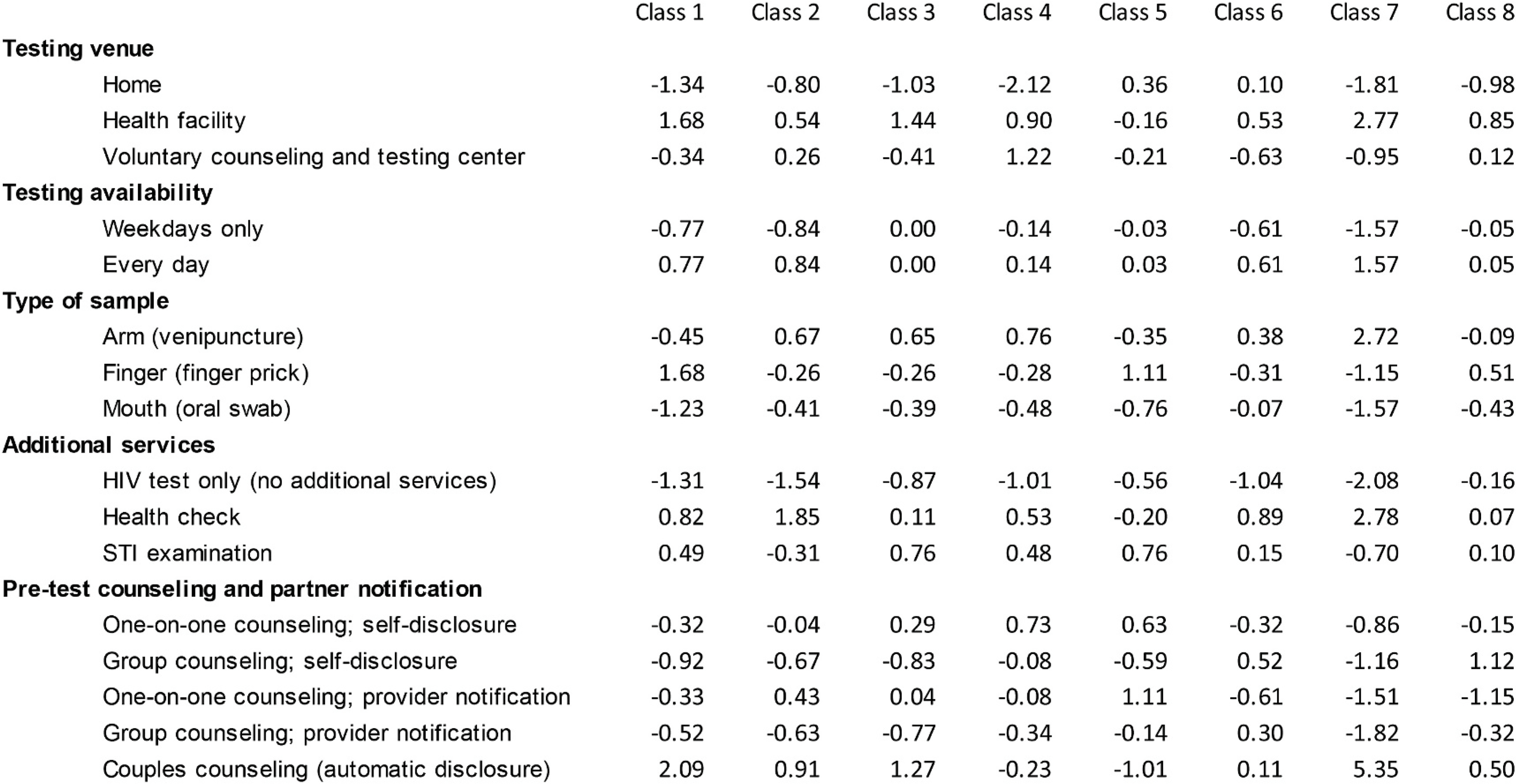
**Panel C.** Parameter estimates for male porters (N = 440).

**Figure:**
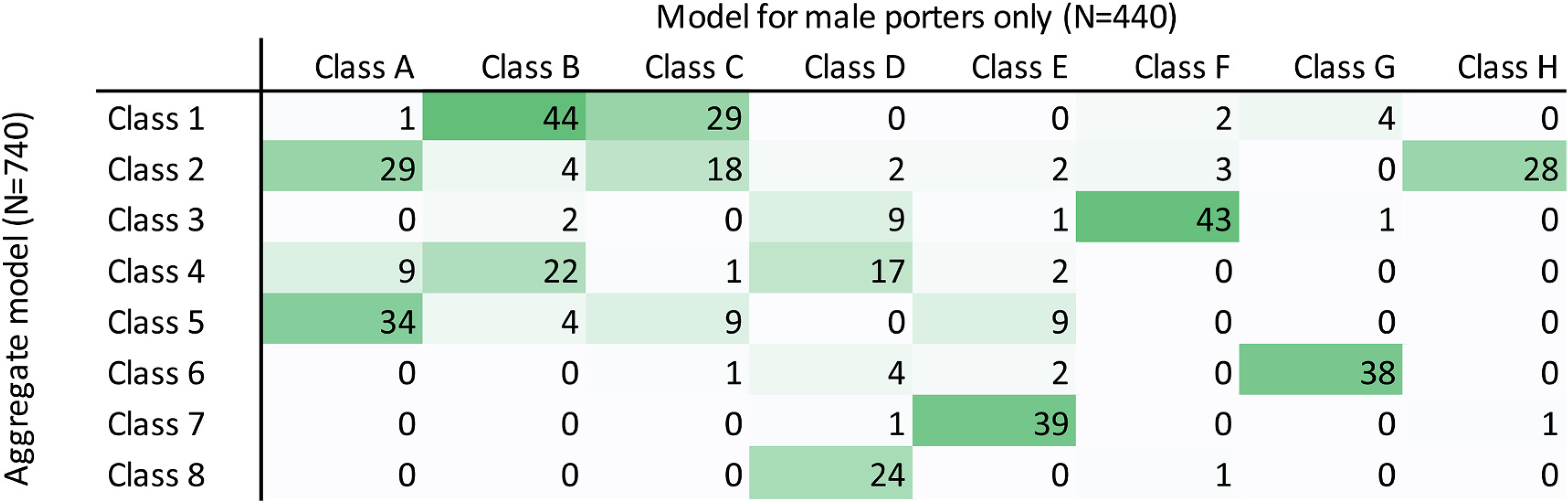
**Panel D.** Concordance between class membership predictions from gender-specific (N = 440 male porters only) vs. aggregate (N = 740) latent class models

## Appendix 1. Estimates from gender-specific models and strong concordance between class membership predictions from gender-specific vs. aggregate models

**Notes:** Estimates are based on gender-specific RELCL models with the same specification as the aggregate model presented in Table 2 and Fig. 3. Class membership predictions are based on modal class membership probability.
